# The association of uric acid with the development of thyroid nodules: a retrospective cohort study

**DOI:** 10.1186/s12902-022-01119-y

**Published:** 2022-08-08

**Authors:** Yingshi Huang, Zexin Li, Kaiji Yang, Lili Zhang, Chiju Wei, Peixuan Yang, Wencan Xu

**Affiliations:** 1 Health Care Center, the First Affiliated Hospital of Shantou University Medical College, No. 57, Changping Road, Shantou, 515041 China; 2 Department of Radiology, the First Affiliated Hospital of Shantou University Medical College, No. 57, Changping Road, Shantou, 515041 China; 3 Multidisciplinary Research Center, Shantou University, No. 243, Daxue Road, Shantou, 515063 China; 4 Department of Endocrinology, the First Affiliated Hospital of Shantou University Medical College, No. 57, Changping Road, Shantou, 515041 China

**Keywords:** Uric acid, Thyroid nodules, Metabolism, Risk factor, Cohort study

## Abstract

**Background:**

Uric acid was found to have a positive correlation with thyroid nodules in the cross-sectional studies recently. We aimed to conduct a retrospective cohort study to investigate whether uric acid is a risk factor for the development of thyroid nodules.

**Methods:**

We reviewed the data of individuals who attended the medical check-ups in our hospital from 2010 to 2019. A total of 6587 adults without thyroid nodules at baseline were enrolled in this study. Logistic regression with or without restricted cubic spline function was used to investigate the non-linear or linear association between uric acid and thyroid nodules, respectively.

**Results:**

Baseline characteristics showed that subjects mainly consisted of the healthy, young population. After fully adjusting for the potential confounders, such as age, sex, metabolic and inflammatory indicators, hepatic and renal function, a logistic restricted cubic spline regression model suggested that uric acid had a significant association (*P* = 0.028) with the development of thyroid nodules, but the association was not non-linear (*P* = 0.516). The results indicate that the association between them is linear, which was demonstrated by a logistic regression model, in which the odds ratio of uric acid per 100 mmol/L was 1.137 (*P* = 0.004). Age, sex, diastolic blood pressure, fasting blood sugar, and blood monocyte were found to be risk factors for thyroid nodules as well.

**Conclusion:**

Uric acid is an independent risk factor for the formation of thyroid nodules. This finding warrants attention to this risk factor in apparently healthy adults.

## Background

Thyroid diseases nowadays have become common diseases in clinical practice. Graves’ disease and thyroiditis, both of which tend to have explicit symptoms, are easily detected and diagnosed. Thyroid nodules (TNs) of huge size will oppress the ambient tissue and cause a symptom, which will be identified easily as well. However, most TNs are asymptomatic and incidentally found in a routine medical checkup by palpation or ultrasound [1]. It is reported that 4% to 7% of the healthy people are detected to have TNs using palpation, which is the least sensitive methods [2]. The prevalence will reach 67% with a more sensitive method, a high-resolution ultrasound [3]. Due to the high prevalence of TNs, some experts expressed concern about overdiagnosis of TNs and advocated restraint when diagnosing and treating TNs smaller than 1 cm [4, 5]. Furthermore, clinical guidelines have been updated in recent years in order to reduce the overdiagnosis of low-risk TNs [6]. It was reported that approximately 5%-13% of TNs were identified with one or more malignant characteristics by ultrasound, CT, or MRI [7]. Even though the TNs are identified as benign in clinical and imaging, they still have a potential risk for malignancy [8]. Some factors have been demonstrated to increase the risk of incident TNs, including age, female sex, iodine deficiency, and radiation exposure [9]. More risk factors should be explored, due to the high prevalence of TNs and the probability of malignancy.

Uric acid (UA) is the end product of purine catabolism, which is present mostly in the liver, intestines, muscles, and the vascular endothelium. Although UA plays an important biological role as an antioxidant, high levels of UA are associated with many metabolic and non-metabolic diseases. The direct impairment of high UA comes from gout, but it has also been linked with other metabolic disorders, such as insulin resistance, hypertension, and diabetes [10].

There is a close link between metabolic disorders and TNs. A study investigated Chinese people living in a moderate iodine intake area and found that people with metabolic syndrome have higher morbidity of TNs than those without. After correction for the possible confounders, logistic regression showed that waist circumference, high blood pressure, and fasting blood glucose are positively and independently associated with TNs [11]. In a mild-to-moderate iodine-deficient area, a prospective study was performed to assess thyroid volume and TN prevalence in patients with pre-diabetes and type 2 diabetes and found that patients with pre-diabetes (51.3%) or diabetes (61.8%) have a higher percentage of TNs than the control (23.7%). Further analysis suggested that impaired glucose metabolism is a predictor of the higher thyroid size and TNs prevalence [12]. Elevated UA, as one of the indicators of metabolic disorder, was also found associated with TNs in a recent study [13]. However, there is only one cross-sectional study focusing on UA and TNs. More evidence is needed, and the cause and effect need to be elucidated. This study aimed to investigate the effect of UA on the formation of TNs using a retrospective cohort in China.

## Methods

### Study population

We respectively collected the data of the subjects who attended health check-ups in our department in the First Affiliated Hospital of Shantou University Medical College from January 2010 to December 2019. We didn’t collect the data before 2010 or after 2019 because a few of the analyzers were upgraded at these two time points. Subjects resided in a coastal city that has implemented the salt iodization policy since 1993. Seafood products, which contain abundant iodine, accounted for a large part of their diet. Thus, subjects were considered to be iodine-adequate. In our department, clients with severe diseases or clinical symptoms were led to the emergency or outpatient department. Thus, subjects were considered to be healthy or mildly ill. The inclusion criteria were: (1) subjects who have two or more health check-up records; (2) have comprehensive examinations including UA and other metabolism-related indicators, complete blood count, hepatic function, renal function, and thyroid ultrasound. The exclusion criteria were: (1) subjects with thyroid nodules; (2) with a history of thyroid surgery; (3) with suspicious Graves’ disease and thyroiditis identified by thyroid ultrasound; (4) with missing data or outliers. There were 9718 subjects were enrolled in this study according to the inclusion criteria. After exclusion, 6587 TN-free subjects remained for further analysis.

### Measurements and definitions

In our department, individuals were required to be barefooted and least dressed when measuring their body height and weight and were demanded to rest for at least 5—10 min before the blood pressure measurement. For the venous blood obtainment, individuals had to keep fasting from 10 p.m. the night before, and blood was drawn by the laboratorian the next morning (7:30–9:30 a.m.). A complete blood automatic analyzer (XE-2100, Sysmex Corporation, China) was used to test the complete blood count. A biochemical automatic analyzer (Dimension Rxl Max, Siemens Corporation, German) was used to test the hepatic, renal, and metabolic parameters. The thyroid ultrasound was performed with a high-frequency probe by the experienced sonographers. Besides ultrasonic reports, the data we collected included age, sex, metabolic and inflammatory parameters, and hepatic and renal function, all of which were found to be associated with TNs [14–17]. Metabolic parameters included UA, body mass index (BMI), systolic blood pressure (SBP), diastolic blood pressure (DBP), fasting blood sugar (FBS), triglyceride (TG), cholesterol (CHOL), high-density lipoprotein (HDL), and Low-density lipoprotein (LDL). Inflammatory parameters were the traditional ones, such as white blood cell (WBC), lymphocyte (LY), neutrophil (NE), and monocyte (MO). Hepatic function was represented by alanine transaminase (ALT), aspartate aminotransferase (AST), and total bilirubin (TB), and renal function was represented by creatinine (Cr).

This is a retrospective cohort study. Each enrolled subject had at least two records of health check-up. The first record was collected as the baseline, and the last one was collected for the observation of the development of TNs. A period between the first and last records was defined as follow-up time. The body mass index (BMI) was defined as weight divided by the square of height (kg/m^2^). TN disease was defined as any solid and nodular lesion, which was detected by ultrasound to be different from the adjoining parenchyma in the thyroid gland [1].

### Statistical analysis

R software (version 4.0.2)was used for data analysis. In the analysis of the baseline characteristics, continuous data were presented as median (interquartile range, IQR) due to the non-normal distribution, and dichotomous data were presented as number (percentage). Normal distribution of the Continuous data was estimated by Kolmogorov–Smirnov test. Logistic restricted cubic spline (RCS) regression was used to investigate the non-linear association of UA with TNs. To fit a better RCS model, we explore RCS models with knot number from three to seven and chose a model with the lowest Akaike information criteria (AIC). Except for the non-linear association, as sensitivity analysis, we used logistic regression to detect the linear association of UA with TNs as well. In both non-linear and linear models, confounders included were age, sex, body mass index, systolic blood pressure, diastolic blood pressure, uric acid, fasting blood sugar, triglyceride, cholesterol, low-density lipoprotein, high-density lipoprotein, white blood cell, neutrophil, lymphocyte, monocyte, alanine transaminase, aspartate aminotransferase, total bilirubin, and creatinine. Follow-up time, the interval between the baseline and observation time, was different among subjects, a potential confounder, and adjusted as well. *P* < 0.05 was considered to have a statistical significance.

## Results

### Baseline characteristics of the study subjects

The baseline characteristics of the subjects were presented in Table 1. A total of 6587 subjects were enrolled in this study. The population was composed mainly of youth with a median age of 36 (IQR: 27, 45). Among them, 41.3% (2718 subjects) were female. Body mass index, systolic blood pressure, diastolic blood pressure, fasting blood sugar, triglyceride, cholesterol, low-density lipoprotein, high-density lipoprotein, white blood cell, neutrophil, lymphocyte, monocyte, alanine transaminase, aspartate aminotransferase, total bilirubin, and creatinine, taken together, showed a predominance of health population in our study, according to their median and IQR within the reference range. However, the third quartile of UA was 425 mmol/L, suggesting high levels of UA in the population in this area..

**Table 1:** Baseline characteristics of the study subjects

	**Overall**
**n**	6587
**Age (years)**	36 [27, 45]
**Females**	2718 (41.3)
**Body mass index (kg/m**^**2**^**)**	22.5 [20.6, 24.5]
**Systolic blood pressure (mmHg)**	119 [111, 128]
**Diastolic blood pressure (mmHg)**	76 [70, 83]
**Uric acid (mmol/L)**	350 [274, 425]
**Fasting blood sugar (mmol/L)**	5.10 [4.80, 5.49]
**Triglyceride (mmol/L)**	0.96 [0.68, 1.44]
**Cholesterol (mmol/L)**	4.80 [4.20, 5.43]
**Low-density lipoprotein (mmol/L)**	2.92 [2.44, 3.46]
**High-density lipoprotein (mmol/L)**	1.28 [1.08, 1.53]
**White blood cell (10**^**9**^** cells/L)**	6.46 [5.50, 7.65]
**Neutrophil (10**^**9**^** cells/L)**	3.46 [2.83, 4.25]
**Lymphocyte (10**^**9**^** cells/L)**	2.35 [1.95, 2.81]
**Monocyte (10**^**9**^** cells/L)**	0.39 [0.31, 0.48]
**Alanine transaminase (U/L)**	26 [18, 35]
**Aspartate aminotransferase (U/L)**	21 [18, 26]
**Total bilirubin (mmol/L)**	14.0 [10.7, 17.6]
**Creatinine (mg/dl)**	78 [64, 92]

Data are presented as median (interquartile range) and number (percentage)

### Association of uric acid with the development of thyroid nodules

**Fig. 1:**
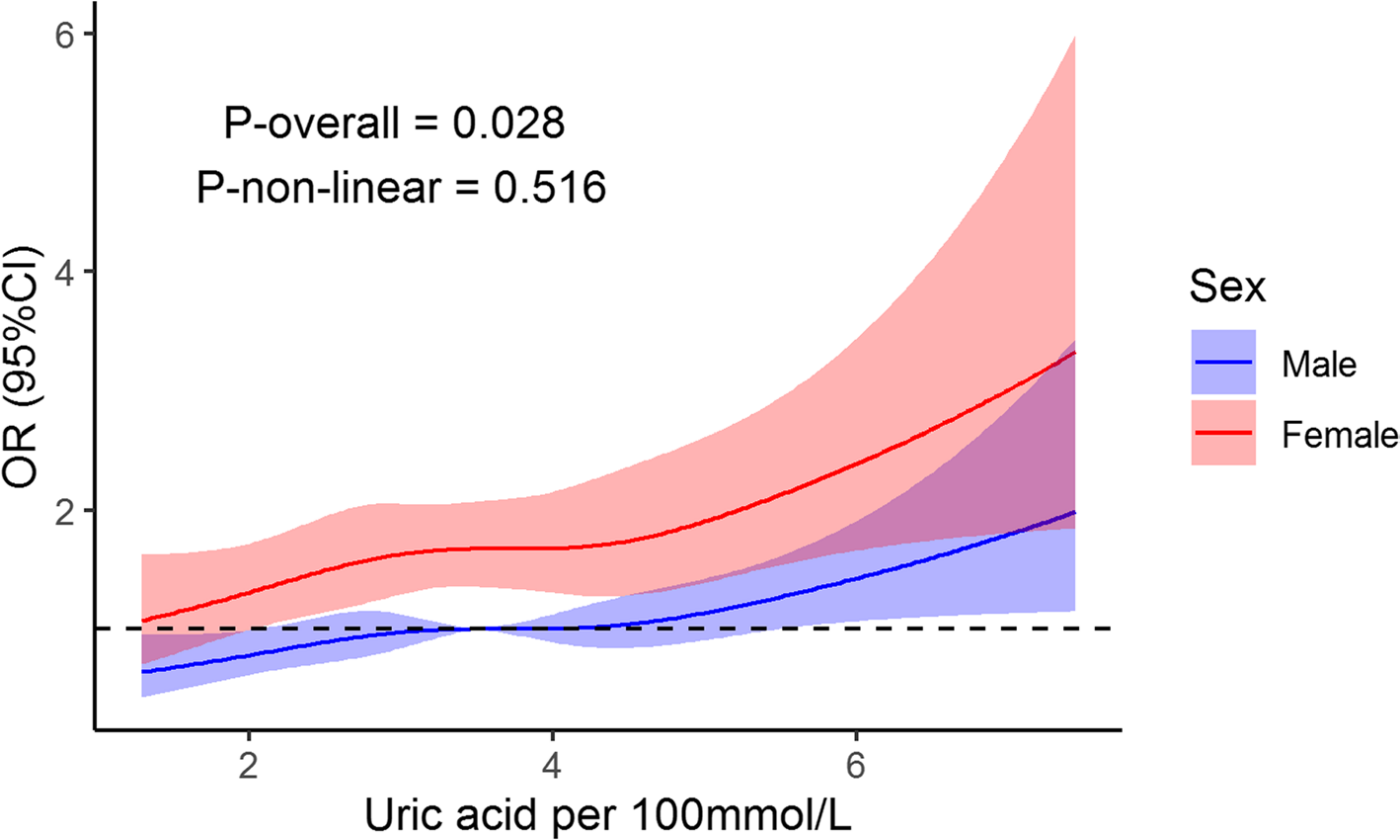
Association between uric acid and thyroid nodules. Logistic restricted cubic spline regression with three knots was performed to explore the non-linear association between uric acid and thyroid nodules. Variables in this model included uric acid per 100 mmol/L (restricted cubic spline), age, sex, body mass index, systolic blood pressure, diastolic blood pressure, uric acid, fasting blood sugar, triglyceride, cholesterol, low-density lipoprotein, high-density lipoprotein, white blood cell, neutrophil, lymphocyte, monocyte, alanine transaminase, aspartate aminotransferase, total bilirubin, creatinine, and follow-up time. *P*-overall < 0.05 suggests a significant association between uric acid per 100 mmol/L and thyroid nodules. *P*-non-linear < 0.05 suggests a significant non-linear association between uric acid per 100 mmol/L and thyroid nodules

The median follow-up time was 37.3 (IQR: 14.5, 61.8) months, and 1345 subjects developed TNs at the observation time, with an incidence rate of 114 per 1000 inhabitants per year. We used logistic regression with UA per 100 mmol/L as a restricted cubic spline (RCS) variable to explore the non-linear association of UA with TNs. To fit a better RCS model, we explore RCS models with knot number from three to seven, and the model with 3 knots had the lowest AIC of 5945 was chosen. As Fig. 1 shows, along with the increase of UA, the odds ratio increased (*P* = 0.028). The *P* for non-linear association is the insignificance of 0.516, indicating a linear association between UA and TNs. Besides, from Fig. 1, we can observe that females had a higher risk to develop TNs than males, also observed in Table 2 (OR: 1.675, CI:1.354, 2.073). Other risk factors for TNs included age (OR: 2.133, CI: 1.916, 2.374), diastolic blood pressure (OR: 1.137, CI: 1.003, 1.29), fasting blood sugar (OR: 1.056, CI: 1.006, 1.109), monocyte count (OR: 1.142, CI: 1.01, 1.292) (Table 2). Figure 1 shows that females had a higher increasing rate of OR of TNs with the increase of UA, but unexpectedly, no significant interaction (*P* = 0.692) between sex and UA was detected.

**Table 2:** Risk factors for thyroid nodules in the logistic restricted cubic spline regression model

	**Odds ratio (95% CI)**	***P* value**
**Age**	2.133 [1.916, 2.374]	< 0.001
**Females**	1.675 [1.354, 2.073]	< 0.001
**Diastolic blood pressure**	1.137 [1.003, 1.29]	0.044
**Fasting blood sugar**	1.056 [1.006, 1.109]	0.027
**Monocyte**	1.142 [1.01, 1.292]	0.034

A restricted cubic spline model with three knots was performed to explore the non-linear association between uric acid and thyroid nodules. Variables in this model included uric acid per 100 mmol/L (restricted cubic spline), age, sex, body mass index, systolic blood pressure, diastolic blood pressure, uric acid, fasting blood sugar, triglyceride, cholesterol, low-density lipoprotein, high-density lipoprotein, white blood cell, neutrophil, lymphocyte, monocyte, alanine transaminase, aspartate aminotransferase, total bilirubin, creatinine, and follow-up time.

*CI* Confidence interval.

Except for uric acid per 100 mmol/L, which was presented in Fig. 1, variables with a *P*-value < 0.05 were presented in this table

Since the RCS model suggested a linear association between UA and TNs, we used logistic regression to detect the effect size of UA on TNs. As Table 3 shows, odds ratio of UA per 100 mmol/L was 1.137 (CI: 1.043, 1.240, *P* = 0.004). Other risk factors found in this model were similar to those in the RCS model, such as age (OR: 1.043, CI: 1.037, 1.049), female (OR: 1.694, CI: 1.376, 2.086), diastolic blood pressure (OR: 1.010, CI: 1.000, 1.020), fasting blood sugar (OR: 1.081, CI: 1.008, 1.160), monocyte count (OR: 2.205, CI: 1.110, 4.573) (Table 3).

**Table 3:** Risk factors for thyroid nodules in the logistic regression model

	**Odds ratio (95% CI)**	***P* value**
**Age**	1.043 [1.037, 1.049]	< 0.001
**Females**	1.694 [1.376, 2.086]	< 0.001
**Diastolic blood pressure**	1.010 [1.000, 1.020]	0.044
**Fasting blood sugar**	1.081 [1.008, 1.160]	0.03
**Monocyte**	2.205 [1.110, 4.573]	0.033
**Uric acid per 100 mmol/L**	1.137 [1.043, 1.240]	0.004

Logistic regression was performed to explore the linear association between uric acid and thyroid nodules. Variables in this model included uric acid per 100 mmol/L, age, sex, body mass index, systolic blood pressure, diastolic blood pressure, fasting blood sugar, triglyceride, cholesterol, low-density lipoprotein, high-density lipoprotein, white blood cell, neutrophil, lymphocyte, monocyte, alanine transaminase, aspartate aminotransferase, total bilirubin, creatinine, and follow-up time.

*CI* Confidence interval.

Variables with a *P*-value < 0.05 were presented in this table

## Discussion

In this retrospective cohort study, we investigated the association between UA and the development of TNs. In two logistic regression models, with and without restricted cubic spline function, UA was found to be positively associated with the development of TNs.

A large cross-sectional study, conducted by Yao Liu et al., estimated the prevalence of TNs and investigated its association with metabolism-related parameters, especially UA on 67,781 residents (33,020 men, 34,761 women) in northwest China. After adjusting for BMI, SBP, TC, TG, and FBS, serum UA appeared to be negatively associated with TNs in males over 30 years old, but positively associated with TNs in both males under 30 years old and females over 30 years old [13]. In our previous study focusing on the metabolic syndrome and TNs, in multivariate logistic regression, we surprisingly found that not only the components of metabolic syndrome but also UA were positively associated with TNs [18].

Although the cross-sectional analysis showed the independent and positive relation between UA and TNs, we still cannot determine the cause-effect relationship between UA and the formation of TNs. We carried out a retrospective cohort study and found that a higher UA in baseline will increase the risk to develop a TN disease in the future. No previous evidence has demonstrated UA can promote TNs formation or the growth of thyroid cells. There is a possible explanation that might interpret this result. A study supplied evidence of UA directly inducing insulin resistance (IR) in vivo and in vitro. Increased UA levels may affect insulin receptor substrate 1 and protein kinase B, both of which are import links of insulin signaling, to induce IR. Besides, the reactive oxygen species pathway is also involved in the process of UA-inducing IR [19]. Another study found that UA can induce the activation of NLRP3 inflammasome, leading to insulin signaling impairment, and causing IR [20]. IR can cause hyperinsulinemia, namely higher insulin levels in the circulating system. In the case of IR, cells in the liver, muscle, fat, and other tissue do not use insulin efficiently to help transfer the blood sugar into cells. High blood sugar will stimulate the islet β cells to excrete more insulin into the blood, leading to hyperinsulinemia. It is reported that excessive insulin can stimulate the growth of thyroid cells by not only promoting DNA synthesis but also modulating protein synthesis and accumulation. Intracellular cyclic AMP (cAMP) may play an important role in this process [21]. Thus, increased UA may promote the formation of TNs by inducing IR and hyperinsulinemia.

High levels of TSH result in the presence of TNs. Because TSH stimulates TSH receptors/cAMP/PKA pathways in thyroid cells, thereby activating CREB and controlling thyroid cell differentiation and proliferation [22]. Nevertheless, no evidence proves that people with higher UA have higher TSH. A retrospective study analyzed the association between UA and thyroid hormone in 48,526 subjects and found that, in regression analysis, there was a linear relationship between uric acid levels and both free triiodothyronine (FT3) and free thyroxine (FT4), but not with TSH [23]. A population-based study including 405 participants detected that UA is positively associated with FT4 but not TSH [24]. Based on these evidence, we concluded that UA does not promote the formation of TNs by the effect of TSH, which was not measured in our study.

Epidemiological studies have found that thyroid nodules are more prevalent as people age [25, 26]. With increasing age, the thyroid may suffer degenerative changes which lead to diffuse compensatory hyperplasia of the thyroid, eventually resulting in nodules [27]. Females are more predisposed to suffer from TNs, because estrogen, rich in females, can promote the growth of thyroid progenitor cells and stem cells [28]. TNs are closely related to metabolic syndrome components such as abdominal obesity, hypertension, dyslipidemia, and hyperglycemia [29, 30]. Our study has a similar result that diastolic blood pressure and fasting blood sugar were good predictors for TNs. A large-scale study including 133,698 adults demonstrated that systemic inflammation may be involved in the development of TNs, with the monocyte-to-high-density lipoprotein cholesterol ratio strongly associated with the presence and size of TNs in a logistic regression model [31]. We as well found a close relationship between TNs and inflammation, represented by blood monocyte count.

Although we have demonstrated the positive association between UA and TNs after adjusting a wide range of potential confounders, there are limitations to this study due to its retrospective format. Some potential confounders, such as comorbidities and genetic information, were not collected. However, the advantage of this study is that it is first demonstrated UA is a risk factor for the formation of TNs. The results need a further demonstration by a prospective design, accounting for more bias and confounders.

## Conclusions

UA is an independent risk factor for the development of TNs. Active intervention may benefit patients with high UA in terms of the prevention of TNs.

## Data Availability

The datasets generated and/or analyzed during the current study are not publicly available due to the containing information that could compromise research participant privacy/consent but are available from the corresponding author on reasonable request.

## Abbreviations

BMI: Body mass index
CI: Confidence interval
FBS: Fasting blood sugar
FT4: Free thyroxine
IR: Insulin resistance
OR: Odds ratio
RCS: Restricted cubic spline
SBP: Systolic blood pressure
TG: Triglyceride
TNs: Thyroid nodules
TSH: Thyrotropin
UA: Uric acid
